# Apoptosis inducing factor (AIF) mediates lethal redox stress induced by menadione

**DOI:** 10.18632/oncotarget.12562

**Published:** 2016-10-11

**Authors:** Hesti Lina Wiraswati, Emilie Hangen, Ana Belén Sanz, Ngoc-Vy Lam, Camille Reinhardt, Allan Sauvat, Ariane Mogha, Alberto Ortiz, Guido Kroemer, Nazanine Modjtahedi

**Affiliations:** ^1^ Equipe 11 labellisée Ligue Nationale contre le Cancer, Centre de Recherche des Cordeliers, Paris, France; ^2^ INSERM, U1138, Paris, France; ^3^ Gustave Roussy Cancer Campus, Villejuif, France; ^4^ Faculty of Medicine, Université Paris-Saclay, Kremlin-Bicêtre, France; ^5^ Institut Teknologi Bandung (ITB), Bandung, Indonesia; ^6^ Laboratory of Nephrology, IIS-Fundacion Jimenez Diaz UAM and REDINREN, Madrid, Spain; ^7^ INSERM, U1030, Villejuif, France; ^8^ Metabolomics and Cell Biology Platforms, Gustave Roussy Cancer Campus, Villejuif, France; ^9^ Université Paris Descartes, Sorbonne Paris Cité, Paris, France; ^10^ Université Pierre et Marie Curie, Paris, France; ^11^ Pôle de Biologie, Hôpital Européen Georges Pompidou, AP-HP, Paris, France; ^12^ Department of Women's and Children's Health, Karolinska Institute, Karolinska University Hospital, Stockholm, Sweden

**Keywords:** mitochondria, quinone metabolization, oxidative stress, protein arylation, Autophagy

## Abstract

Mitochondrial apoptosis inducing factor (AIF) is a redox-active enzyme that participates to the biogenesis/maintenance of complex I of the respiratory chain, yet also contributes to catabolic reactions in the context of regulated cell death when AIF translocates to the cytosol and to the nucleus. Here we explore the contribution of AIF to cell death induced by menadione (2-methyl-1,4-naphtoquinone; also called vitamin K3) in conditions in which this pro-oxidant does not cause the mitochondrial release of AIF, yet causes caspase-independent cell killing. Depletion of AIF from human cancer cells reduced the cytotoxicity of menadione. This cytoprotective effect was accompanied by the maintenance of high levels of reduced glutathione (GSH), which are normally depleted by menadione. In addition, AIF depletion reduced the arylation of cellular proteins induced by menadione. This menadione-triggered arylation, which can be measured by a fluorescence assay, is completely suppressed by addition of exogenous glutathione or N-acetyl cysteine. Complex I inhibition by Rotenone did not mimic the cytoprotective action of AIF depletion. Altogether, these results are compatible with the hypothesis that mitochondrion-sessile AIF facilitates lethal redox cycling of menadione, thereby precipitating protein arylation and glutathione depletion.

## INTRODUCTION

Naturally occurring quinone derivatives represent a wide range of compounds that not only are implicated in the regulation of crucial cellular processes such as mitochondrial respiration but have also been successfully used as cytotoxic agents (e.g. daunorubicin, doxorubicin, geldanamycin and mitomycin C) for anticancer therapy [1-4]. Quinones, including those that are known for their anti-cancer properties, are classified as bioreductive compounds as their activity depends on their one- or two-electron reduction catalyzed by various cellular quinone reductases [5]. The one-electron reduction of the compound results in the formation of an unstable semiquinone radical that reacts immediately with the oxygen leading to the generation of reactive oxygen species (ROS). While the cytotoxicity of many quinone-related drugs has been attributed to their rapid redox cycling associated with the generation of an intense oxidative stress due to the liberation of ROS, for others the cell death-inducing potential has been linked to their arylation capacity, i.e. the potential to covalently modify cellular constituents such as cysteine of proteins and reduced glutathione (GSH), through the formation of quinone-thiol Michael adducts [6, 7]. There is also a third class of cytotoxic quinones for which the molecular basis of the lethal action is not well established, likely because they act both through redox cycling and the depletion of cellular thiols with the consequent collapse of the anti-oxidant defense [6, 7]. One such a compound is menadione (2-methyl-1,4-naphtoquinone; also called vitamin K3) that is explored, at elevated concentrations, for its anti-cancer potential [6, 8-10]. Two-electron reduction of menadione is responsible for the formation of a stable hydroquinone compound, without the generation of semiquinone as free radical intermediates, perhaps as an attempt to detoxify menadione [6, 7].

In various cell types, flavoproteins have been implicated in both one-electron and two-electron types of reduction pathways and it has been proposed that competition between these enzymatic pathways dictates the sensibility or resistance of the cell to the cytotoxic action of the quinone drug. For instance, while NADPH-cytochrome P-450, NADPH-cytochrome P-450 reductase b5 reductase or NADPH-ubiquinone oxidoreductase have been implicated in the catalysis of one-electron reduction, the flavoprotein NAD (P) H : quinone oxidoreductase 1 (NQO1; DT diaphorase) was described as the enzyme responsible for the two-electron reduction of quinone drugs [6, 7, 11-13]. Many reports indicate that in addition to a small fraction of NQO1 that resides in the mitochondrial matrix[14, 15], additional mitochondrial flavoproteins, such as lipoamide dehydrogenase, could have diaphorase activities and participate to the reduction of quinone compounds [15-17]. The flavoprotein apoptosis inducing factor (AIF), which operates in the intermembrane space of the mitochondrion and is required for the optimal activity of respiratory chain complexes [18, 19], could also act as an NADH : quinone reductase [20, 21]. This suggestion is in agreement with a previous report from our laboratory showing that AIF-knocked out mouse embryonic stem cells exhibit resistance to menadione [22].

Here, we explored the impact of AIF expression on the cytotoxic action of menadione and other quinone compounds. We found that the mitochondria-localized AIF stimulates the lethal redox stress induced by menadione, underscoring its potential to participate in quinone metabolism.

## RESULTS AND DISCUSSION

### AIF depletion protects cells against killing by quinone-related drugs

**Figure 1:**
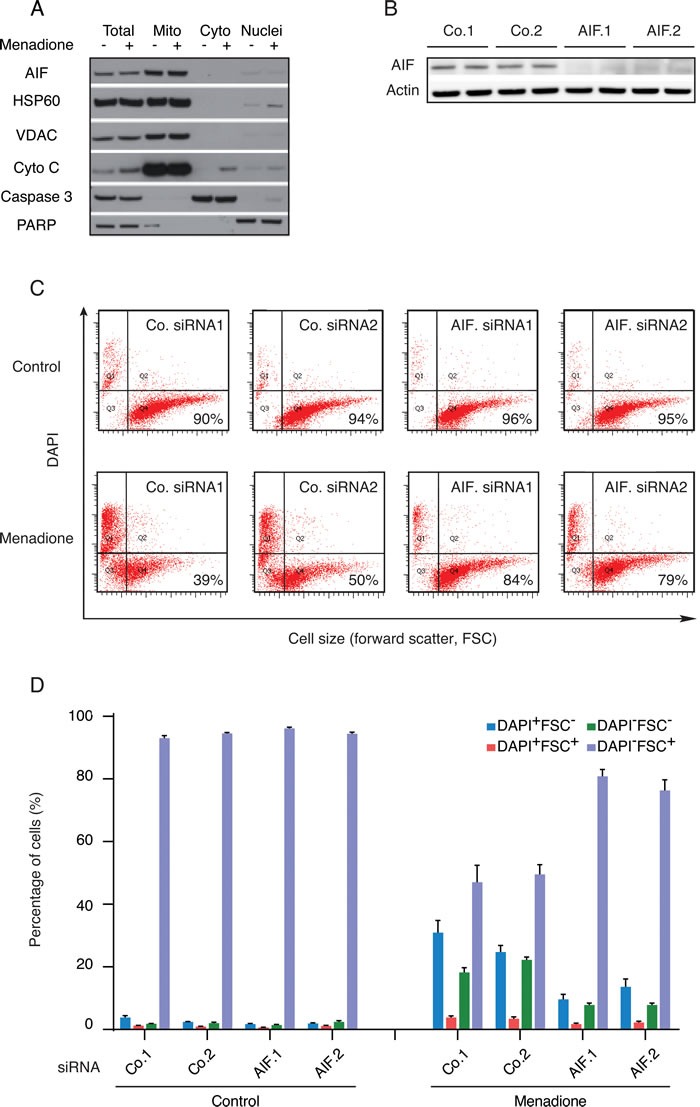
Impact of AIF expression on the cytotoxicity of menadione **A.** After 3h treatment with (+) or without (−) 50μM menadione, the subcellular localization of AIF in U2OS cells was checked by immunoblot in the indicated fractions, using antibodies directed against proteins localized in mitochondria (HSP60, VDAC and cytochrome *c*), cytosol (caspase-3) or nuclei (PARP). **B.** Extracts of U2OS cells (duplicates) subjected to the transfection with AIF-specific (AIF.1 and AIF.2) or control (Co.1 and Co.2) siRNA were analyzed by immunoblot for the abundance of AIF. Actin was used as a loading control. **C. D.** U2OS cells transfected with two distinct control siRNAs (Co.1 and Co.2) or two distinct, non-overlapping siRNAs targeting AIF (siRNA AIF.1 and AIF.2) were treated with 50μM menadione or the solvent (Control) for 3h and drug-induced cell death was quantified by flow cytometric assessment (pictograms shown in C; histograms shown in D) of DAPI uptake (DAPI positivity) and forward light scatter (FSC) analysis that allows for the identification of apoptotic cells according to their reduced size (low FSC). Data are expressed as mean values ± SEM.

**Figure 2:**
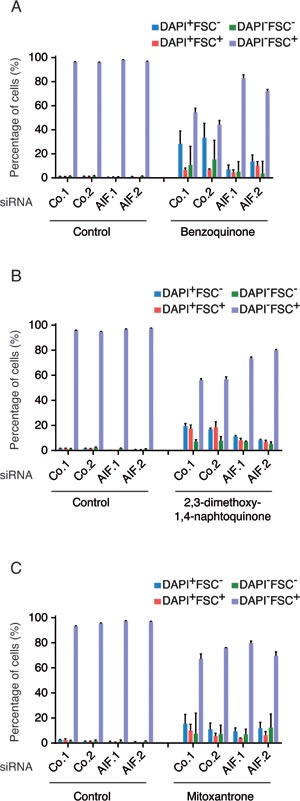
Impact of AIF expression on the cytotoxicity of members of Quinone-derived drug family **A.**-**C.** U2OS cells transfected with two distinct control siRNAs (Co.1 and Co.2) or two distinct, non-overlapping siRNAs targeting AIF (siRNA AIF.1 and AIF.2) were treated with 120μM benzoquinone (BZQ) for 3h (A); 20 μM 2,3-dimethoxy-1,4-naphtoquinone (DMNQ) for 48h (B) or 6 μM mitoxantrone (MTX) for 19h (C) or the solvent (Control). Drug-induced cell death was quantified by flow cytometric assessment (histograms) of DAPI uptake (DAPI positivity) and forward light scatter (FSC) analysis that allows the identification of apoptotic cells according to their reduced size (low FSC). Data are expressed as mean values ± SEM.

Dose-dependency and kinetic experiments (Supplemental Figure 1A and 1B) revealed that human osteosarcoma U2OS cells cultured with menadione (50 μM for 3h) died rapidly in a caspase-independent manner (Supplemental Figure 1C) without exhibiting a significant translocation of AIF from mitochondria to the cytosol (Figure 1A). In these conditions, AIF depletion by two distinct, non-overlapping siRNAs (Figure 1B) reduced the menadione-induced cell death as compared to that observed in cells treated with control siRNAs. Hence, AIF depletion inhibited the menadione-stimulated reduction of cell size (measured with the forward scatter, FSC), as well as the permeabilization of the plasma membrane to the vital dye 4′,6-diamidino-2-phenylindole (DAPI) (Figure 1C and 1D). These results extend and confirm the observation that AIF deletion in mouse embryonic stem cells can confer protection against menadione-mediated cytotoxicity [22]. The cytoprotective effect of AIF depletion was also observed for other quinone compounds (for dose finding and kinetic experiments see Supplemental Figure 1D to 1H) such as benzoquinone (Figure 2A) and 2,3-dimethoxy-1,4-naphtoquinone (Figure 2B), but not mitoxantrone (Figure 2C). Thus, deletion or depletion of AIF confers cytoprotection against several quinone-based cytotoxic agents.

### The cytotoxic function of AIF is not sensitive to rotenone

**Figure 3:**
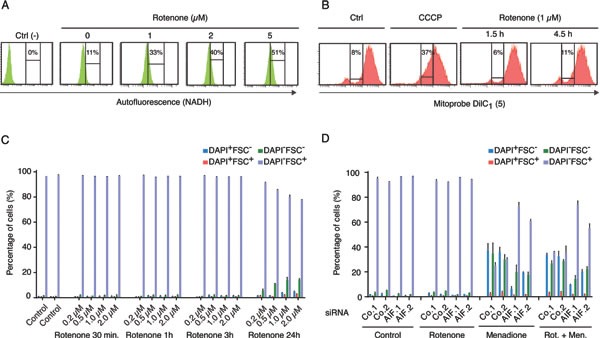
Effect of respiratory chain complex I inhibition on Menadione-induced cytotoxicity **A.** Dose-dependent rotenone-induced respiratory chain complex I inhibition was monitored by flow cytometry, after excitation with a UV laser. The augmentation of cell fluorescence reflects the accumulation of NADH in the treated cells. The NADH-dependent autofluorescence of cells incubated with 1 to 5 μM of rotenone for 4.5 h was compared to that of untreated cells (0 μM) and negative control cells (Ctrl) permeabilized with 70% ETOH before analysis. **B.** The mitochondrial membrane potential of cells incubated with 1 μM rotenone for 1.5 h or 4.5 h was monitored by flow cytometry, using the MitoProbe Dil C_1_(5), and compared to that of control cells untreated (Ctrl) or treated with the protonophore CCCP, which dissipates the mitochondrial transmembrane potential. For each condition, the percentage of cells showing a reduced Dil C_1_(5) incorporation is mentioned. **C.** The effect of rotenone on cell survival was quantified by flow cytometric assessment of DAPI uptake (DAPI positivity) and forward light scatter (FSC) analysis, after incubation with 0 to 2μM of Rotenone for the indicated times. **D.** Effects of AIF knockdown, combined or not with rotenone treatment (1 μM for 4.5 h), on menadione-induced (50 μM for 3 h) cytotoxicity was analyzed after transfection with two distinct control siRNAs (Co.1 and Co.2) or two distinct, non-overlapping siRNAs targeting AIF (siRNA AIF.1 and AIF.2). Cell death was monitored by flow cytometric assessment of DAPI uptake (DAPI positivity) and forward light scatter (FSC) analysis. Data are expressed as mean values ± SD.

AIF is required for optimal function of the respiratory chain complex I [23, 24] and has actually been reported to possess an NADH : ubiquinone oxidoreductase activity that is sensitive to rotenone [21], knowing that rotenone is also a well-established inhibitor of complex I [25]. In conditions (1 μM of rotenone, 4.5 h hours of treatment), in which rotenone increased the cellular content of NADH (measured as the autofluorescence of live cells after excitation at 355 nm, at an emission wavelength of 450 nm, and that is abolished upon cell permeabilization[26]), likely as a consequence of the inhibition of the NADH oxidase activity of complex I (Figure 3A), yet failed to reduce the mitochondrial transmembrane potential (measured with the 1,1′,3,3,3′-hexamethylindodicarbo-cyanine iodide, abbreviated DilC_1_(5)) (Figure 3B) and to decrease cellular viability (Figure 3C), rotenone did not alter menadione-induced cell killing (Figure 3D). Moreover, rotenone did not modify the cytoprotective effect of AIF depletion with respect to menadione (Figure 3D). This kind of epistatic analysis suggest that in human cancer cells, the rotenone-sensitive functions of AIF are not involved in its cytotoxic activity in the context of menadione-mediated killing.

### Effects of AIF on glutathione and menadione metabolism

**Figure 4:**
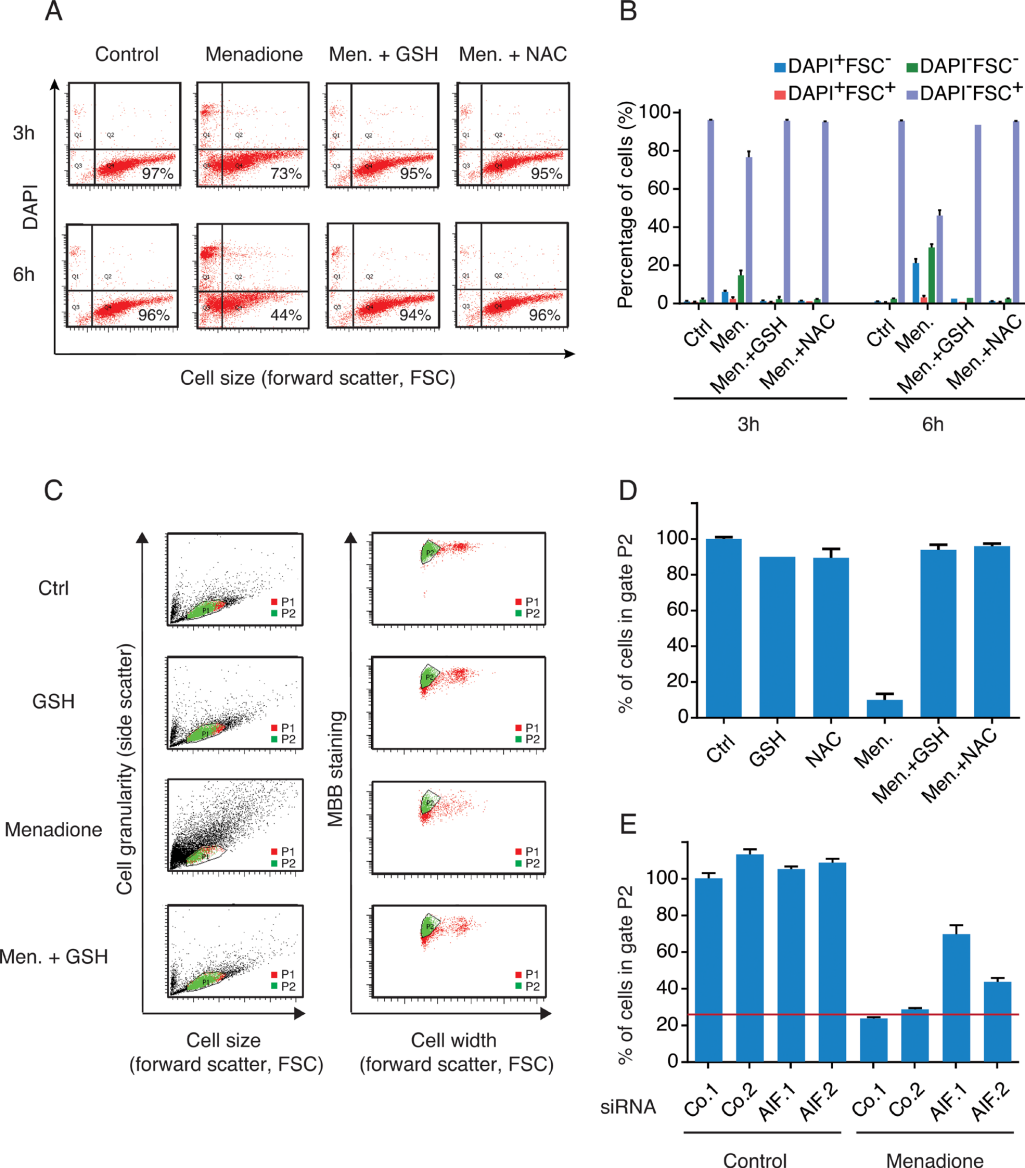
The loss of GSH levels in menadione-treated cells correlates with the expression level of AIF **A.**, **B.** Effect of exogenous antioxidants on menadione-induced death was evaluated by incubating U2OS cells, for 3h or 6h, with 50μM of menadione in the absence or presence of GSH (5 mM) or NAC (5 mM). Cell death was quantified by flow cytometric assessment (pictograms are shown in A and histograms in B) of DAPI uptake (DAPI positivity) and forward light scatter (FSC) analysis that allows the identification of apoptotic cells. **C.**, **D.** A cytofluorimetric analysis combined with the use of the thiol-reactive probe monobromobimane (MBB) was set up to measure levels of reduced glutathione in cells treated with menadione (pictograms are shown in C and histograms D). After menadione treatment, in absence or presence of exogenous antioxidants (GSH or NAC), live cells (Topro3 negative), exhibiting size and granularity parameters similar to control untreated cells (gate P1), were analyzed for their staining with MBB (gate P2). Cell width assessment by forward light scatter (FSC) analysis was used to discriminate between singlet cells and aggregates. For each treatment condition, the percentage of cells stained with MBB (gate P2) was quantified (D). **E.** The effect of AIF knockdown on the levels of GSH was monitored, as described in (C and D), after transfection with two distinct control siRNAs (Co.1 and Co.2) or two distinct, non-overlapping siRNAs targeting AIF (siRNA AIF.1 and AIF.2) and culture with 50 μM of menadione for 3h. Data are expressed as mean values ± SD.

**Figure 5:**
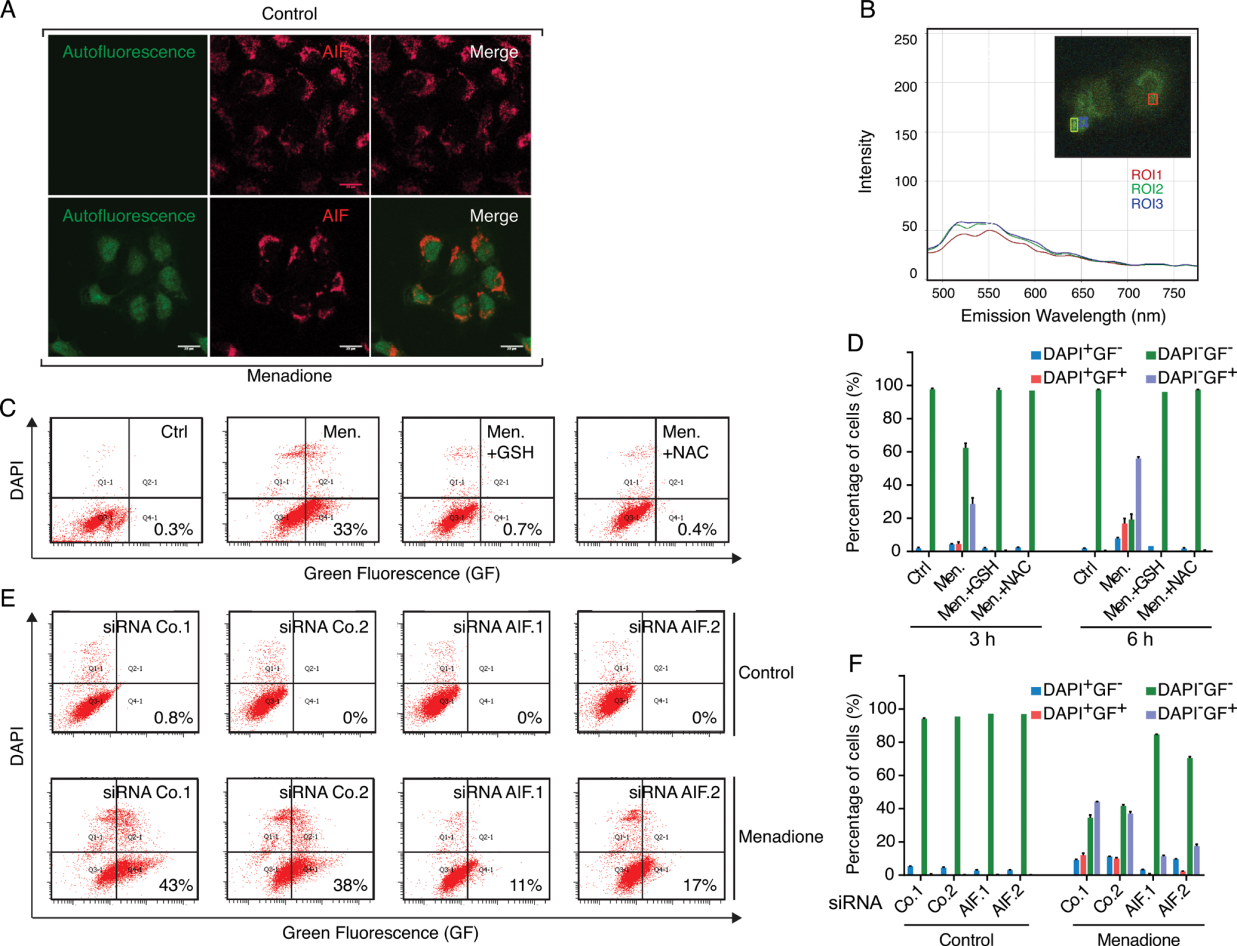
The metabolization of fluorescent menadione-cysteinyl group conjugates correlates with AIF expression levels **A.** Microscopic analysis of U2OS cells revealed that, compared to control conditions (cells treated with the solvent), the incubation with 50 μM menadione for 3 h provoked the appearance of a diffuse cellular fluorescence that resisted to the fixation/permeabilization protocol. The mitochondrial localization of AIF, both in control and menadione-treated cells, was revealed by indirect immunofluorescence, using an anti-AIF rabbit polyclonal antibody and an Alexafluor 647-conjugated secondary anti-rabbit antibody (AIF red staining). Individual and merged images show that in menadione-treated cells, AIF is not released from the mitochondrion and the diffuse distribution of menadione-induced autofluorescence is maximal in the nuclear compartment. **B.** Emission spectra and intensity analyses of the fluorescence produced in menadione-treated cells were evaluated by microscopy. The insert corresponds to the menadione-treated cell that was imaged by fluorescence microscopy (Zeiss) and squares on the image correspond to distinct regions of interest (ROI1 to to ROI3) that were evaluated for fluorescence spectra. **C. D.** The formation of fluorescent menadione-cysteinyl group conjugates (green fluorescence, GF) was monitored by flow cytometric analysis of U2OS cells incubated for 3h or 6h with 50 μM menadione, in the absence or presence of exogenous antioxidants GSH (5 mM) or NAC (5 mM). Analyses of the pictograms (C) and histograms (D) reveal that treatments with both exogenous GSH and NAC inhibit the formation of the fluorescent menadione-cysteinyl group conjugates in menadione-treated cells. **E. F.** After transfection with two distinct control siRNAs (Co.1 and Co.2) or two distinct, non-overlapping siRNAs targeting AIF (siRNA AIF.1 and AIF.2), cells were submitted to menadione treatment (50μM) for 3 h and then analyzed, as in C and D, for the formation of fluorescent menadione-cysteinyl group conjugates. Analyses of the pictograms (E) and histograms (F) reveal that the depletion of AIF inhibits the formation of the fluorescent menadione-cysteinyl group conjugates in menadione-treated cells. DAPI staining was used for monitoring of death-induced membrane permeabilization. Data are expressed as mean values ± SD.

**Figure 6:**
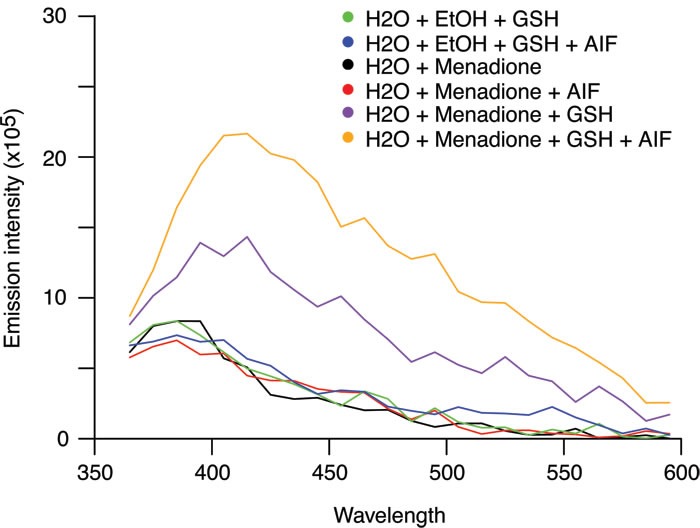
Impact of recombinant AIF on the arylating capacity of menadione To assess the arylating activity of menadione and evaluate the capacity of AIF to stimulate the formation of thioester bonds between menadione and GSH, menadione (250 μM) was directly added to an aqueous solution of GSH (100mM) in the absence or presence of recombinant purified AIF (40 μg/ml). Emission spectra were analyzed after excitation at 340 nm. The combination of menadione with GSH (purple line) produced fluorescence. The addition of recombinant AIF stimulated the arylating capacity of menadione that could be measured by the enhancement of the fluorescence (orange line). No fluorescence was detected for menadione, GSH alone or the combination of menadione with AIF alone.

The cytotoxic activity of menadione was fully abolished by supplementation of cells with reduced glutathione (GSH) or the GSH precursor N-acetyl cysteine (NAC) (Figure 4A and 4B). Menadione provoked a decrease in the endogenous levels of GSH (as measured with the specific dye Monobromobimane, MBB) among cells with a still normal shape and hence unaltered side scatter (SSC) and forward scatter (FSC) characteristics (Figure 4C). This loss of endogenous GSH was fully inhibited by addition of exogenous GSH or NAC (Figure 4D), and was partially reduced by depletion of AIF (Figure 4E). We observed that addition of menadione to cells caused the appearance of a green fluorescence that withstood cell pemeabilization and fixation (Figure 5A and 5B), an experimental procedure that eliminates small soluble molecules not engaged in complex structures. The emission spectrum of this menadione-induced fluorescent signal (Figure 5B) was rather different from that of NADH, which peaks at 450 nm and is only detectable in live non-permeabilized cells (Figure 3A). Simultaneous detection of membrane permeability with DAPI and the menadione-induced fluorescent signal revealed that the latter appeared before the cells lost viability (Figure 5C). Addition of either GSH or NAC to menadione-treated cells abolished the fluorescent signal (Figure 5C, 5D). Moreover, AIF depletion strongly reduced the menadione-induced fluorescence (Figure 5E, 5F). Altogether, these results underscore the strong correlation existing between intracellular depletion of the reducing agent GSH and the surge of the menadione-induced fluorescent signal. Both GSH depletion and the menadione-induced fluorescence are facilitated by the expression of AIF. This kind of menadione-induced fluorescence has been reported to result from the arylation of cellular proteins including nuclear histones [27, 28]. One argument in favor of this possibility is the observation that exogenous GSH, which is not cell membrane-permeable (contrary to its ethyl ester that enters cells), protected cells from the cytotoxic effect of menadione and abolished the fluorescent signal (Figure 5C and 5D). In the past, studying an endothelial cell death model, Chang et al. [29] suggested that the protective effect of GSH could be explained by its capacity to conjugate with menadione outside of the cell and reduce the cellular uptake of the unconjugated form of the quinone. By using GSH as a thiol donor in an *in vitro* acellular fluorometric assay [28], we confirmed that the formation of conjugates between menadione and GSH led to the appearance of a fluorescent arylation product (Figure 6). Thus, fluorescence spectra analysis revealed that the addition of menadione to the GSH solution sufficed to generate a fluorescence that was undetectable with menadione or GSH alone (Figure 6). Within the same assay, we evaluated the impact of the recombinant AIF protein on the arylating capacity of menadione. The addition of AIF resulted in the enhancement of the fluorescence signal of the menadione-GSH conjugate, confirming that AIF stimulated the arylating capacity of menadione (Figure 6). It is worth mentioning that no fluorescence could be detected for menadione combined with AIF alone (Figure 6). In conclusion, experiments in cell-free systems indicate that AIF interacts with menadione *in vitro* and this interaction is independent from the presence of additional proteins or the cellular context.

### Concluding remarks

AIF is a redox active enzyme, and NADH oxidase [30] that has first been suggested to participate in anti-oxidant reactions as a ROS scavenger, meaning that its absence may sensitize cells to oxidant-induced stress [31]. In contrast with this interpretation, however, AIF potentiates cell death by pro-oxidants [22] including menadione (2-methyl-1,4-naphtoquinone) that has been explored for its therapeutic potential under the name of vitamin K3 [6]. AIF has previously been shown to act as an NADH : quinone reductase [20, 21], renewing our interest in the effects of AIF on quinone-induced cell killing. Here, we confirm in several assays that depletion of AIF reduces cell killing by menadione (and other quinones). In the past, it was suggested that the cytoprotective effect of AIF knockdown is explainable by the attenuation of AIF mitochondrio-nuclear translocation in menadione-treated cells [32]. Here, we show for the first time that the cytoprotective effect of AIF knockdown observable in menadione-treated human cancer cells, is not due to the attenuation of its apoptotic function in the nucleus but rather related to the downregulation of its mitochondrial activity that impacts on arylation reactions mediated by the quinone compound. Cytoprotection against menadione by AIF depletion is also accompanied by the avoidance of GSH depletion. One plausible scenario to explain this effect of AIF depletion is the following. AIF depletion reduces the deleterious redox cycling of menadione, hence avoiding GSH depletion, which is a usual consequence of menadione treatment. The conserved GSH pool then acts as an antioxidant against menadione (which indeed directly interacts with menadione to form a menadione-GSH conjugate) [33], thereby avoiding the lethal arylation of cellular proteins. It should be noted that rotenone, which reportedly inhibits the NADH : ubiquinone oxidoreductase activity of AIF in the bacterial cell context [21], failed to reduce cellular killing by menadione. This finding either suggests that rotenone is unable to interfere with the quinone-reductase activity of AIF in the mammalian cell context or that the precise AIF-catalyzed redox reaction that is inhibited by rotenone is irrelevant to the interaction of AIF with menadione. Irrespective of this incognita, however, it appears clear that, in the mitochondrial intermembrane space, AIF actively contributes to menadione-induced cell killing through an effect on cellular redox biochemistry. At the molecular level, it is unclear how the mitochondria-localized AIF can fulfill additional enzymatic functions that are unrelated to its activity in the regulation of the respiratory chain complexes. For instance, it would be interesting to see whether the monomer-dimer structural transition of AIF, which is known to be triggered upon binding with its cofactor NADH [34], or in response to oxidative stress [35], could also have an impact on the equilibrium between its respiratory chain-regulating and quinone-reductase functions. One could also speculate that upon exposure to quinone compounds, quinone-reductase activities of additional flavoproteins other than AIF such as NQO1 or lipoamide dehydrogenase could also be modified, thus impacting on the metabolism of the quinone molecule in a compound-dependent or tissue-specific manner. Moonlighting functions [36] have already been described for the mitochondrial matrix-localized lipoamide dehydrogenase, which is known for its capacity for both one and two-electron reduction of quinone compounds [16, 17, 37]. Reportedly, in conditions of ischemia-reperfusion, acidification of the mitochondrial matrix alters the oligomeric structure of lipoamide dehydrogenase and consequently diminishes its dehydrogenase function while favoring the appearance of its diaphorase activity [17].

Irrespective of these incognita, the present results underscore the capacity of mitochondrion-localized AIF to impact redox metabolism, thereby favoring the lethal action of quinone derivatives.

## MATERIALS AND METHODS

### Reagents and antibodies

Chemicals were as follows: Menadione (Sigma Aldrich), 1,4-Benzoquinone (BZQ; USP), 2,3-Dimethoxy-1,4-naphthoquinone (DMNQ, Sigma Aldrich), Mitoxantrone dihydrochloride (MTX, Sigma Aldrich), Carbonyl cyanide 3-chlorophenylhydrazone (CCCP; Tocris), Monobromobimane (MBB; Thermo Fischer Scientific), L-glutathione reduced (GSH; Sigma Aldrich), *N*-Acetyl-L-cysteine (NAC; Sigma Aldrich), 4′,6-Diamidino-2-Phenylindole, Dihydrochloride (DAPI; Thermo Fischer Scientific), Rotenone (Sigma Aldrich), Z-VAD.fmk, (BACHEM); DilC_1_ (5) (Thermo Fischer Scientific). ECL chemiluminescence detection kit (GE Healthcare).

Antibodies against the following proteins were used: actin (mouse mAb; CHEMICON); AIF (mouse mAB; Santa Cruz; rabbit pAB; Cell Signaling); Hsp60 (mouse mAb; Stressgen); caspase 3 (rabbit pAB; Cell Signaling); PARP (rabbit pAB; Cell Signaling); VDAC (rabbit pAB; Cell Signaling); Cytochrome C (mAB BD Bioscience).

### Cell culture and transfection

Human Osteosarcoma U2OS cells (ATCC n°HTB-96) were cultured at 37°C and 5% CO_2_ in Dulbecco's modified Eagle's medium (DMEM; Life Technologies) supplemented with 10% heat-inactivated fetal bovine serum (FBS) (Life Technologies) and 1% penicillin/streptomycin. siRNA transfections were performed using Lipofectamine-2000 reagent (Life Technologies) by following manufacturer's procedure. For RNA interference experiments the following siRNA sequences were used: for negative control: Co.1: CCG UGC UCC UGG GGC UGG G [dT][dT]; Co.2 : AUG CAG AAC UCC AAG CAC G [dT][dT]; for human AIF: AIF.1: GGG CAA AAU CGA UAA UUC U[dT][dT]; AIF.2: GCA UGC UUC UAC GAU AUA A [dT][dT] (Delettre 2006).

### Cell fractionation

2.5 × 10^6^ U2OS cells were incubated with ETOH (control) or with 50 μM Menadione for three hours and then processed for sub-cellular fractionation using a cell fractionation kit and protocol provided by the manufacturer (Mitosciences). Finally, after adding 1% SDS, equal volumes of each subcellular fraction (which correspond to equal number of cells) were subjected to immunoblot analyses using the specified antibodies.

### Immunoblotting

For whole extract preparation, cells were harvested, washed three times with ice-cold PBS (8.1 mM Na2HPO4, 135 mM NaCl, 1.5 mM KH2PO4, 2.7 mM KCl), lysed with 1% SDS, boiled, sonicated and then stored at -80°C. Proteins contained in the lysate were quantified (Bio-Rad DC protein assay) and then resolved by SDS-PAGE (NUPAGE; Invitrogen) after boiling in 1xSB (2% SDS, 10% glycerol, 62.5 mM Tris-HCl, pH 6.8, 100 mM dithiothreitol). For immunoblot analysis, SDS-PAGE-resolved proteins were transferred onto nitrocellulose membrane (Biorad). After transfer, membranes were blocked for 1 h by incubation with 5% nonfat milk dissolved in TBST buffer (10 mM Tris-HCl pH 8.0, 150 mM NaCl, 0.05% Tween 20), and then for further 16 h at 4°C with the specified primary antibody diluted in the same incubation mixture supplemented with 0.02% Na-azide. The membrane was then washed three times in TBST buffer before incubation with a secondary antibody conjugated to horseradish peroxidase. Antibody binding was revealed by chemiluminescence using an ECL detection kit (GE Healthcare).

### Fluorescence microscopy analyses

For the microscopic analysis of autofluorescence, cells were grown on coverslips for 16hr, treated with 50 μM for 3 h before being fixed with 4% PFA. After mounting with Fluoromont-G mounting medium (Southern biotech), coverslips were observed with a Zeiss confocal microscope and fluorescence spectra induced by menadione treatment was evaluated in multiple regions of interest (ROI) within the same cell.

For indirect immunofluorescence analysis, cells were fixed on coverslips for 15 min with 4% PFA and then permeabilized for 5 min with Triton 0.2% at room temperature. AIF protein was detected by incubation with an anti-AIF pAB (1:100 dilution in PBS / 1% BSA / 2% goat serum) followed by Alexafluor-647-conjugated goat anti-rabbit (Life Technologies). DNA was stained with Hoechst 3342 or TO-PRO-3 (Life Technologies). Sample slides were then mounted using the reagent Fluoromont-G (Southern Biotech) and observed by confocal microscopy (LSM-510; Carl Zeiss Microimaging) equipped with an X63 objective.

### Flow cytometry

The following probes were used for the cytofluorometric assessment of cell death parameters: 4′,6-Diamidino-2-Phenylindole, Dihydrochloride (DAPI, 3 μM) for the plasma membrane permeabilization, and Mitoprobe 1,1′,3,3,3′-hexamethylindodicarbo-cyanine iodide (DilC_1_(5), 50 nM) for the assessment of mitochondrial membrane potential dissipation. Monobromobimane (MBB, 50μM) was used for the quantification of the reduced glutathione (GSH) content of the cell. The detection of the fluorescent menadione-cysteinyl group conjugates was achieved using the green (FITC) channel. The determination of the NADH-dependent autofluorescence was achieved after excitation, with a UV laser, while gating on, TO-PRO-3-negative, live cells. Cytofluorometric analyses were realized using a FACS Aria Cell sorter or an LSRII flow cytometer (BD Biosciences) equipped with FACSDiva software (version 6.1.2; BD Biosciences).

### Recombinant AIF protein expression and purification

Human AIF protein (residues 121-613) was overexpressed in E. coli by applying an overnight IPTG induction protocol (0.5 mM IPTG at 20°C). The recombinant AIF protein that contained both N- and C-terminal His-tags was purified by Nickel-affinity chromatography and gel filtration, concentrated to 4 mg/ml and finally stored at -80°C ([38].

### Fluorometric assessment of reduced glutathione (GSH) arylation by menadione

Freshly prepared menadione stock (50 mM) was directly added to black 96 wells plates with clear bottom (Greiner) that contained the aqueous fluorophore assessment mixture composed of 100 mM GSH. Immediately after, the fluorescence of the sample was analyzed using a SpectraMax^®^ i3 (Molecular Devices) using the indicated excitation and emission wavelengths. Collected data were analyzed with the R software (https://www.r-project.org/).

## SUPPLEMENTARY MATERIALS FIGURE


