# Supplementary material for: Apoptosis inducing factor (AIF) mediates lethal redox stress induced by menadione
Source: Oncotarget. 2016 Oct 11;7(47):76496–507. doi: 10.18632/oncotarget.12562 (PMC5363526; doi:10.18632/oncotarget.12562)
Supplement: Supplementary file 1 [file oncotarget-07-76496-s001.pdf]

Apoptosis inducing factor (AIF) mediates lethal redox stress induced by menadione

Supplementary Material

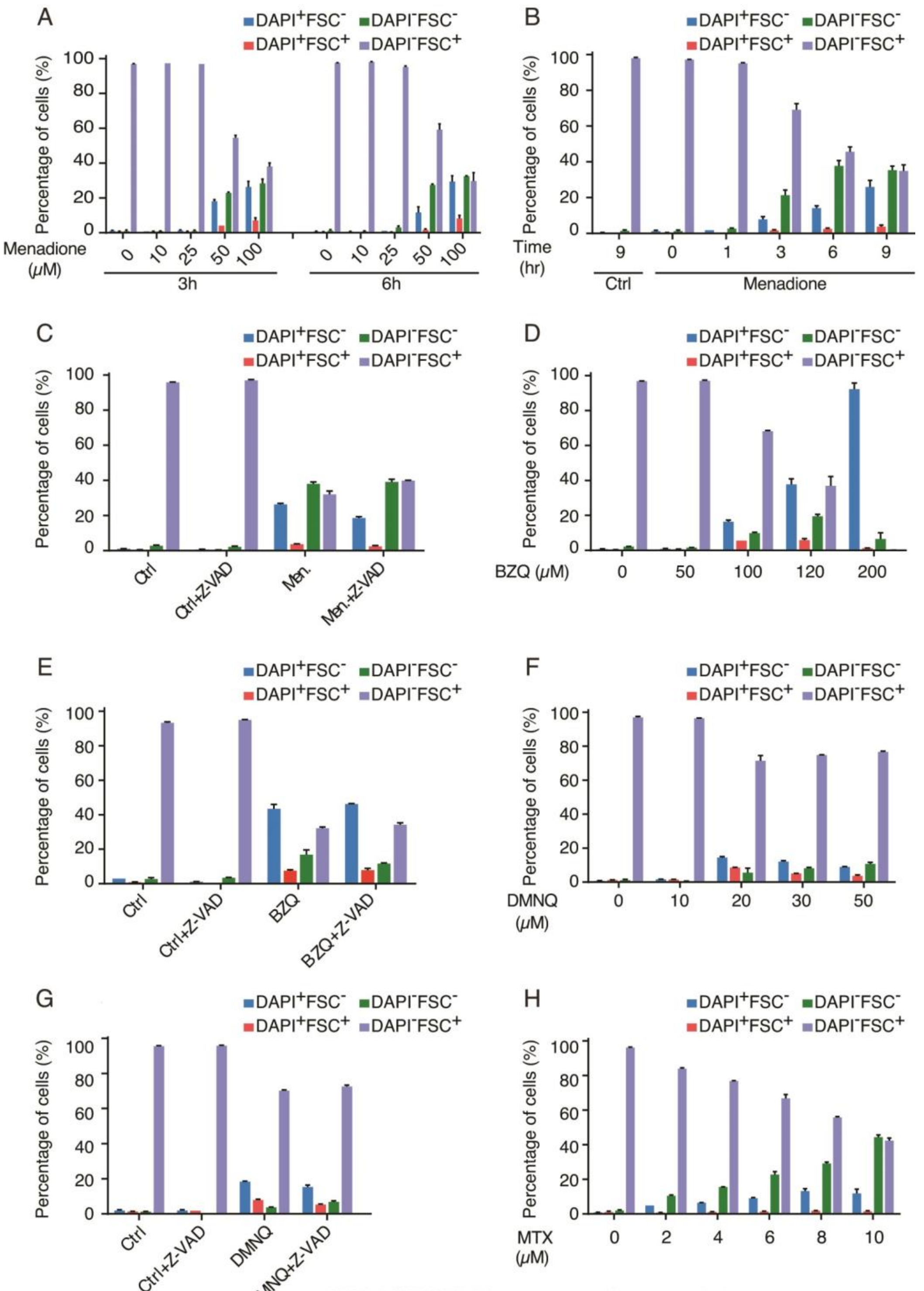

WIRASWATI Figure supplemental 1

Supplemental Figure 1: Analysis of U2OS cell death induced by quinone drugs. (A-C) U2OS cells were treated with the indicated doses of menadione for 3 or 6 h (A) or with 50 µM of menadione for the indicated times (B). The caspase-independency of menadione-induced death of U2OS cells was evaluated by incubating the cells with 50 µM of menadione in absence or presence of 50 µM of the pan-caspase inhibitor Z-VAD-fmk (C). Cell death was quantified by flow cytometric assessment of DAPI uptake (DAPI positivity of the cells with permeabilized membranes) and forward light scatter (FSC) analysis that allows the identification of apoptotic cells according to their reduced size (low FSC). (D) (E) Cells were treated with the indicated doses of BZQ for 3h (D). The caspase-independency of BZQ-induced death of U2OS cells was evaluated by incubating the cells with 120 µM of BZQ in absence or presence of 50 µM of the pan-caspase inhibitor Z-VAD-fmk (E). BZQ-induced cell death was monitored by flow cytometry as in A. (F) (G) U2OS cells were treated with the indicated doses of DMNQ for 48 h (F). The caspase-independency of DMNQ-induced death of U2OS cells was evaluated by incubating the cells with 20 µM of DMNQ in absence or presence of 50 µM of the pan-caspase inhibitor Z-VAD-fmk (G). DMNQ-induced cell death was monitored by flow cytometry as in A. (H) U2OS cells were treated with the indicated doses of MTX for 19 h (H). MTX-induced cell death was monitored by flow cytometry as in A. Data are expressed as mean values ± SD.
